# Method optimization of protein and nucleic acid extraction from skin tape strip samples for biomarker analysis

**DOI:** 10.3389/fmed.2026.1665962

**Published:** 2026-05-25

**Authors:** Erika L. Boarder, Jessica L. Moore, Kyra A. Richardson, Angelina Volkova, Danielle Guttierez, Cynthia D. Timmers, Susan H. Smith

**Affiliations:** 1 Incyte Corporation, Wilmington, DE, United States; 2 Discovery Life Sciences, Huntsville, AL, United States

**Keywords:** atopic dermatitis, biomarkers, dermatology, noninvasive biomarker, protein extraction, proteomics, tape stripping, transcriptomics

## Abstract

**Objective:**

Skin tape stripping is a minimally invasive, skin-specific method for biomarker collection that has gained increasing interest in dermatologic research. However, the lack of standardized protocols for protein and nucleic acid extraction from tape strips has limited its broader application. This study aimed to establish robust and broadly compatible protocols for biomarker extraction from tape strip samples to enable reliable downstream proteomic and transcriptomic analyses.

**Methods:**

We systematically evaluated key technical parameters influencing biomarker recovery, including collection and storage conditions, buffer composition, and lysis strategies. Protein and nucleic acid extraction protocols were optimized for maximum yield and quality across a range of downstream applications, including immunoassays and transcriptomic profiling.

**Results:**

The optimized protein extraction protocol demonstrated improved total yield and compatibility with multiple assay platforms. A parallel nucleic acid extraction method yielded high-quality RNA suitable for gene expression analyses. Notably, we found that disease-relevant biomarkers were detectable in the most superficial tape strips, indicating that fewer total strips may be sufficient for effective analysis.

**Conclusion:**

We present practical and standardized methods for protein and nucleic acid extraction from skin tape strip samples. These protocols address current technical barriers and support broader implementation of tape stripping as a biomarker collection strategy in clinical dermatology and related fields.

## 1 Introduction

Atopic dermatitis (AD), a chronic inflammatory skin disease with rising global prevalence, has served as a prime model for studying cutaneous immune dysregulation and barrier dysfunction (1, 2). Traditional insights into its pathophysiology have relied extensively on skin biopsies, which, although highly informative, are invasive and present significant limitations in clinical trials, especially for pediatric cohorts and longitudinal studies due to associated pain, risk of scarring, and potential for infection, not to mention the negative impact on patient recruitment.

As a minimally invasive alternative, tape stripping (TS) has emerged as a robust technique for sequential removal of the stratum corneum (SC), allowing sampling of corneocytes and associated biomolecules from the upper epidermis. First described in the mid-20th century and originally employed for morphologic studies (3–5), TS sampling involves the subsequent application of adhesive tape discs to collect biologic material from the stratum corneum that contains skin-specific biomarkers (6, 7). Varying reports have shown this technique can be used to measure protein, DNA, RNA, lipids, or microbial content (8–15). However, several questions remain for translational scientists incorporating tape strip samples into clinical research, including knowing how many tapes (quantity or “depth”) are required for the biology of interest. Nonetheless, the method’s simplicity and patient tolerability make it particularly suited for routine clinical settings, repeated assessments, and use in vulnerable populations. Moreover, recent advances in analytical technologies have significantly enhanced the sensitivity of TS-based assays. Multiplex proteomic platforms, such as the MESO Scale Discovery and Olink proximity extension assay, now enable quantification of a broad array of cytokines and chemokines from a single tape sample (16). In clinical studies, TS has revealed distinct immunologic profiles in AD skin—demonstrating elevated T helper cell type 2 (Th2)-associated cytokines [e.g., interleukin (IL)-13, C-C motif chemokine ligand (CCL) 17/thymus and activation-regulated chemokine (TARC)], as well as IL-1β and IL-8, correlating with disease severity and transepidermal water loss (TEWL) (17–20). Compared with skin biopsies, TS have been shown to better capture epidermal cytokines and pruritus-related biomarkers [e.g., IL-31, transient receptor potential cation channel V3 (TRPV3)], while also detecting critical elements of the skin barrier such as filaggrin (FLG) degradation products and lipid alterations (21–23). Moreover, TS-based transcriptomic profiling has unveiled differential gene expression patterns between lesional and nonlesional AD skin, sometimes identifying epidermal genes not detected in full-thickness biopsies (11). This suggests that TS and biopsy methods may provide both overlapping and complementary data.

In summary, alternative methods for skin biomarker collections, including TS sampling, continue to gain attention in recent years. Their ease of use, cost effectiveness, and presumed compatibility with downstream molecular assays highlight skin TS sampling as a viable alternative to full thickness punch biopsies in clinical and translational dermatology studies for many applications (11, 24). It offers significant advantages in safety, repeatability, and molecular accessibility, particularly for capturing dynamic epidermal immune and barrier biomarkers.

This manuscript aims to promote the use of TS sampling as an alternative biomarker collection method by exploring the technical considerations, capabilities, and limitations of the TS method in analyzing skin biomarkers, with a goal of developing a versatile and scalable extraction process for both protein and nucleic acids. This effort may enable broader adoption of TS sampling across industry and academic settings and thus provide a less invasive alternative for patients in translational and therapeutic dermatology studies.

## 2 Materials and methods

### 2.1 Study population

Thirty-two healthy donors and 14 individuals diagnosed with AD were enrolled in this study by Sanguine Bioscience (Waltham, MA), following protocols approved by the institutional review board (IRB). Protocols were approved by the Western Copernicus Group IRB and Advarra ethics committee. Patient written consent was obtained prior to sample collection. The group of healthy donors consisted of 19 females, ages 24–59, and 13 males, ages 22–75; AD donors consisted of 10 females, ages 23–67, and 4 males, ages 39–60. Exclusion criteria for this study included use of investigational product in the past 30 days and use of hypochlorite “bleach” bath within 15 days prior to visit.

### 2.2 Sample collections

For healthy donors, a sample consisted of 1–≥ 20 consecutive standard 22-mm diameter D-Squame sampling discs (CuDerm Corporation, Cat. No. D100, Dallas, TX, United States) that were collected from the volar forearm. For AD collections, ≤ 20 consecutive D-Squame sampling discs were applied to active lesions > 10 cm^2^ in size and/or an analogous nonlesional area. After placing each TS sampling disc, consistent pressure was applied for several seconds using a D-Squame Pressure Instrument (CuDerm Corporation, Model D500, Dallas, TX, United States) before removal of the tape disc. Subsequent discs were applied to the same location. The location was marked based on the first tape disc application with a pen or skin-safe marker to allow for consistent placing of subsequent tape discs. Following collection, samples were placed into 2-mL cryogenic tubes (Corning^®^, Cat. No. 430488, Corning, NY, United States), with adhesive side facing inward, and then immediately frozen on dry ice before storage at −70°C. The SquameScan^®^ 850A device (CuDerm Corporation, Cat. No. SS850A, Dallas, TX, United States) was used to assess the total biological material collected on each TS sampling disc by measuring the optical absorption of the disc at 850 nm (infrared light) as previously described (25).

### 2.3 Protein extraction

Protein extraction was performed to evaluate the efficacy of different lysis protocols. Briefly, tapes were cut into four quarters using surgical scissors. A volume of 300 μL of either Radioimmunoprecipitation Lysis and Extraction Buffer (RIPA; Thermo Fisher Scientific, Cat. No. 89900, Waltham, MA, United States) or phosphate-buffered saline (PBS; Thermo Fisher Scientific, Cat. No. 10010023, Waltham, MA, United States), each supplemented with 1 × Halt Protease and Phosphatase Inhibitor Cocktail (PPI; Thermo Fisher Scientific, Cat. No. 78440, Waltham, MA, United States), was added to a 1.5-mL microcentrifuge tube containing 4 tape quarters (1 quarter from each donor) (26, 27).

Samples were then subjected to 1 of the following mechanical lysis conditions adapted from previously published methods. In brief, lysis buffer was added to samples and placed on ice for 30 min (16). Extraction was then carried out by (a) vortexing vigorously for 1 min, (b) sonication in a chilled (2–4°C) ultrasonic water bath (Fisher Scientific, Cat. No. 15-336-106, Waltham, MA, United States) for 15 min (26), (c) shaking at 1,400 rpm for 30 min at 4°C (9, 28), or (d) shaking at 1400 rpm for 16–22 h at 4°C (28).

To test the impact that pooling multiple tapes has on yield, a combination of 1, 2, 4, or 6 tapes were lysed in 500 μL of RIPA buffer containing 1 × PPI. Cells from ≤ 6 tapes were gently scraped into a single tube containing lysis buffer by using a rubber cell lifter (CELLTREAT Scientific Products, Cat. No. 229305, Pepperell, MA, United States). Samples were placed at 4°C with shaking overnight then spun down for 15 min at 15,000 × g. The supernatant was then transferred to a new tube for further testing.

To assess the protein yield across the stratum corneum, tapes were harvested in sets of 2, up to tape 20. Tapes were placed in 2-mL microcentrifuge tubes, in sets of 2, and samples were lysed by shaking in 300 μL of RIPA buffer containing 1 × PPI at 1,400 rpm for 16–22 h at 4°C. Samples were centrifuged at 4°C for 15 min at 15,000 × g, after which the supernatant was then transferred to a new tube.

Protein extraction for Olink Explore HT and mass spectrometry (MS) was performed by scraping 4 tapes, up to tape 20, into 200 μL of M-PER Mammalian Protein Extraction Reagent (Thermo Scientific, Cat. No. 78501, Waltham, MA, United States) containing 1 × PPI (Thermo Fisher Scientific, Cat. No. 78440, Waltham, MA, United States) and 1 μM of ethylenediaminetetraacetic acid (Thermo Fisher Scientific, Cat. No. 78440, Waltham, MA, United States) and shaking overnight at 4°C. Samples were centrifuged at 4°C for 15 min at 15,000 × g, and then the supernatant was transferred to a new tube. Both the pellet and supernatant were saved for further analysis.

### 2.4 Protein analysis

The amount of soluble protein extracted from tape strips was measured by Pierce BCA Protein Assay Kit (BCA; Thermo Fisher Scientific, Cat. No. A65453, Rockford, IL, United States) for all samples except those analyzed using Olink Explore HT and MS, which were quantified using the Pierce 660 Protein Assay Kit (Thermo Fisher Scientific, Cat. No. 22662, Rockford, IL, United States).

Samples were prepared for cytokine analysis using the Meso QuickPlex SQ 120 [Meso Scale Discovery (MSD), Rockville, MA, United States] by combining supernatants from tapes extracted in sets of 2 to create 5 zones, representing tape depths: 1–4, 5–8, 9–12, 13–16, and 17–20. Concentrations after pooling were reconfirmed by BCA assay. Pooled samples were normalized to the same protein concentration and cytokine levels measured on the following human MSD V-Plex pre-configured panels, according to the manufacturer’s instructions: Proinflammatory Panel 1, Cytokine Panel 1, Chemokine Panel 1, and Th17 Panel 1. Data were analyzed using Discovery Workbench software (v4.0, MSD, Rockville, MD, United States). Lower limits of detection (LLOD) for proteins were calculated based on fixed LLOD values provided by the manufacturer.

Supernatants from tapes extracted in sets of 4 were prepared for Olink analysis using the Explore HT panel consisting of > 5,400 proteins (Olink Proteomics, Cat. No. 98101, Uppsala, Sweden). Samples were randomized in R prior to analysis. For Olink Explore HT, neat protocols were used when preparing libraries to increase sensitivity. Libraries were sequenced using an Illumina NovaSeq 6000 (Illumina, NovaSeq 6000, San Diego, CA, United States).

Both the supernatant and pellet were analyzed using liquid chromatography tandem mass spectrometry (LC-MS/MS). Briefly, the soluble extracts were prepared using PreOmics iST 96x sample preparation kits (PreOmics, Cat. No. P.O.00027, Martinsried, Germany). Insoluble pellets were solubilized using 10% sodium dodecyl sulfate and processed using Protifi S-Trap™ Micro Column sample preparation kits (Protifi, Cat. No. K02-micro-160, Farmingdale, NY, United States). Following digestion, peptides were quantified using a Pierce Fluorometric Peptide Assay (Thermo Fisher Scientific, Cat. No. 23290, Rockford, IL, United States). Two hundred nanograms of peptides were fractionated over a 50-cm μPAC column over a 55-min gradient using a Vanquish Neo coupled to an Orbitrap Astral MS (Thermo Fisher Scientific, San Jose, CA, United States). Data were collected in DIA mode with 2 Th windows. Resulting peptides were searched using DIA-NN 1.8.1 and processed in R Studio.

### 2.5 Data analysis

Data describing protein and RNA yields were analyzed by GraphPad Prism version 9.3.1 (GraphPad Software, San Diego, CA, United States). Data are shown as box and whisker plots with whiskers extending from the minimum to maximum values and a line denoting the median. Comparisons between groups were performed using an unpaired, two-tailed *t*-test. Differences were considered statistically significant at *P* < 0.05.

Olink and MS data were processed using RStudio (2024.12.0 Build 467) and the Olink Analyze R package (Olink Proteomics, Uppsala, Sweden). Limits of detection (LOD) for proteins measured using the Olink Explore HT platform were calculated based on fixed LOD values provided by the manufacturer. Differential protein expression analysis for Olink Explore HT data was conducted using the same R package, based on normalized protein expression (NPX) values. Differential expression analysis for MS-based proteomics data was performed using RStudio based on protein group normalized intensity (Log10). For both platforms, statistical comparisons were performed using Olink Analyze statistical analyses (Welch two-sample *t*-test) with significance defined as adjusted *P* < 0.05 and multiple testing correction applied using the Benjamini–Hochberg method (false discovery rate).

### 2.6 RNA extraction and analysis

RNA was extracted from TS samples using the Maxwell RSC Blood DNA Kit (Promega, Cat. No. AS1400, Madison, WI, United States) according to the manufacturer’s protocol titled “Automated purification of total nucleic acid (TNA) from dermatological tapes” (Scientific Applications Note PA675), with minor modifications. Briefly, ≤ 10 TS were sequentially lysed in a buffer composed of 300 μL lysis buffer, 300 μL RNA Lysis Buffer (Promega, Cat. No. Z3051, Madison, WI, United States), 12 μL Homogenization Solution, and 12 μL of 1-thioglycerol (Promega, Cat. No. A208B, Madison, WI, United States). Each tape was vortexed for 10 s and centrifuged briefly to collect the lysate, which was transferred to the subsequent tube containing the next tape. Scrapping was incorporated as needed using a rubber cell lifter (CELLTREAT Scientific Products, Cat. No. 229305, Pepperell, MA, United States). After processing all tapes, 30 μL of Proteinase K was added to the pooled lysate, followed by incubation at room temperature for 20 min. Samples were then processed using the Maxwell RSC Instrument, and RNA eluted in nuclease-free water. Eluates were treated with deoxyribonuclease I (DNase I; Life Technologies, Cat. No. 18068015, Carlsbad, CA, United States) and concentrated using the RNA Clean & Concentrator Kit (Zymo Research, Cat. No. R1015, Irvine, CA, United States). RNA was stored at −80°C until further use.

Every other tape from a single donor was allocated to either the Maxwell-based protocol or a phenol-based extraction method, resulting in 10 TS per method. Tapes were processed identically: each was vortexed for 10 s, briefly centrifuged, and the lysate was sequentially transferred to the tube containing the next tape. For the phenol-based method, tapes were lysed in 600 μL of QIAzol Lysis Reagent (Qiagen, Cat. No. 79306, Hilden, Germany). Following sequential pooling, lysates were incubated at room temperature for 5 min, then 120 μL chloroform was added. Samples were vortexed vigorously for 15 s, incubated for 2–3 min at room temperature, and centrifuged at 12,000 × g for 15 min at 4°C. The aqueous phase was carefully transferred to a new tube, mixed with 1 volume of 70% ethanol, and applied to RNeasy Mini spin column (Qiagen, Cat. No. 74106, Hilden, Germany) for purification according to the manufacturer’s protocol.

To test different Maxwell kits, every other tape from a single donor was divided between the Maxwell RSC Blood DNA Kit (Promega, Cat. No. AS1400, Madison, WI, United States) or the Maxwell RSC simplyRNA Cells Kit (Promega, Cat. No. AS1390, Madison, WI, United States), resulting in 10 TS per method. Tapes were processed identically: each was vortexed for 10 s, briefly centrifuged, and the lysate was sequentially transferred to the tube containing the next tape. For the simplyRNA cells method, tapes were lysed in the manufacturer’s recommended buffer, and all subsequent steps were performed according to manufacturer’s protocol. On-board DNase treatment was performed for the RNA cells method while eluates from the blood DNA method were treated with DNase I (Life Technologies, Cat. No. 18068015, Carlsbad, CA, United States). Final eluates were stored at −80°C until further use.

RNA quality was assessed using an automated microfluidic electrophoresis system (Agilent 4200 TapeStation) with High Sensitivity RNA ScreenTape (Agilent Technologies, Cat. No. 5067-5579, Santa Clara, CA, United States). RNA integrity numbers (RIN) were generated using TapeStation Analysis Software v3.2.

To determine the absolute RNA concentration for each donor (tapes 1–20), a standard curve was generated using serial dilutions of purified total RNA from human skin with known concentrations. RNA quality was validated using the Agilent 4200 TapeStation with High Sensitivity RNA ScreenTape (Agilent Technologies, Santa Clara, CA, United States) to confirm RNA integrity and absence of genomic DNA contamination. RNA concentration was quantified using the Qubit RNA High Sensitivity Assay Kit (Thermo Fisher Scientific, Cat. No. Q32852, Waltham, MA, United States). β2-Microglobulin (B2M) transcript copy numbers obtained by ddPCR were interpolated against this standard curve to calculate the total ng of RNA for each sample.

B2M transcript levels were quantified in RNA extracted from 2 sequential TS regions (tapes 1–10 and tapes 11–20) using the QX200 Droplet Digital PCR System (Bio-Rad Laboratories, Hercules, CA, United States). Complementary DNA (cDNA) was synthesized from total RNA using the SuperScript IV VILO Master Mix (Thermo Fisher Scientific, Cat. No. 11754, Waltham, MA, United States). Droplet digital polymerase chain reaction (ddPCR) reactions included 2 × ddPCR Supermix for Probes (no deoxyuridine triphosphate) and a FAM-labeled TaqMan B2M assay (Assay ID: HS00187842_m1, Thermo Fisher Scientific, Cat. No. 2090144, Waltham, MA, United States). Droplets were generated with the QX200 Droplet Generator, amplified under standard cycling conditions (95°C for 10 min; 40 cycles of 94°C for 30 s and 55°C for 1 min; 98°C for 10 min), and analyzed on the QX200 Droplet Reader.

RNA extracted from TS samples was processed using the Watchmaker RNA Library Prep Kit with Polaris Depletion (Watchmaker Genomics) to enable rRNA and globin transcript reduction while preserving transcriptome complexity. Libraries were prepared according to the manufacturer’s instructions. Sequencing was performed on an Illumina NovaSeq platform using paired-end reads with a target depth of 100 million reads per sample.

RNA-seq FASTQ files were processed using the NextFlow pipeline (v. 3.10). Downstream analysis was conducted in R (v. 4.2). The calcNormFactors function from the edgeR package was used to calculate scaling factors for data normalization. The data were then transformed into counts per million (CPM).

## 3 Results

### 3.1 Optimizing collection and storage to preserve sample integrity

For generalized use in a multisite clinical study, clear and consistent guidance on tape collection and shipping or handling processes is crucial. Variation in storage temperatures and shipping times could have meaningful impact on downstream sample quality and usefulness, thereby limiting the utility of this methodology. As such, we first evaluated the patient experience and challenges with shipment. Traditionally, adhesive tape discs have been collected onto storage cards (CuDerm Corporation, Cat. No. D102, Dallas, TX, United States) prior to freezing or shipment. However, challenges were observed with consistency of tape adherence to these storage cards.

**FIGURE 1:**
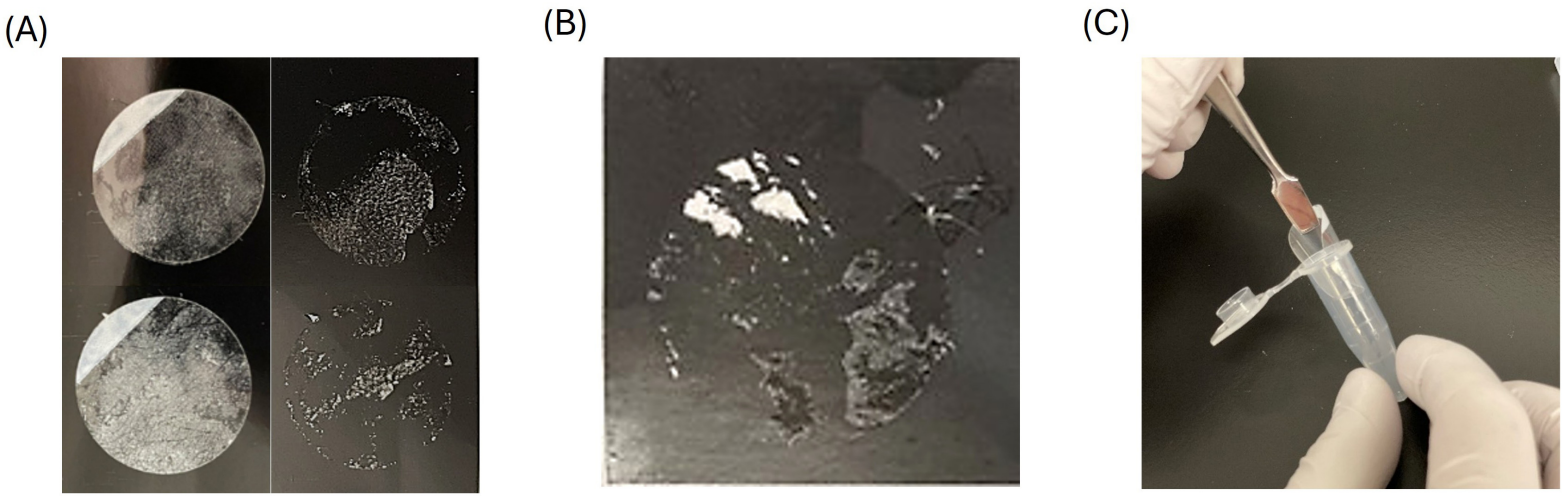
**(A)** Image of two representative tape strips after collection (left panels) and the residual epidermal material remaining on the card after removal (right panels), highlighting the sample loss when applied to D100 storage cards. **(B)** Image of storage card following tape removal, displaying delamination of the card’s surface that has the potential to interfere if carried into downstream workflows. **(C)** Skin tape sample is being placed in tube adhesive side facing inward for long term storage to preserve sample integrity.

(i) Situation 1: Low adherence to storage cards resulting in tapes slipping or falling off the cards. During shipping and handling, sometimes the tapes would slip off the cards such that we no longer had a record of their proper order. This is particularly important if different analyses are planned for specific tape numbers. For example, if tape numbers 2–5 were intended for protein extraction and tapes 6–16 for RNA extraction.
(ii) Situation 2: Low yield due to transfer of biologic material to the storage cards. Adhesive TS collected on D120 standard storage cards (Clin and Derm) demonstrated visible loss of epidermal material, as evidenced by substantial material remaining on the card surface following tape removal. Although epidermal coverage appeared consistent across the tape surface at the time of collection, incomplete retention of material during storage led to considerable variability in how much biological material remained on the tape surface (Figure 1A). This was not an isolated occurrence but was observed across multiple donors, introducing inconsistencies that could significantly impact large-scale studies.
(iii) Situation 3: In certain cases, the tape adhesive bonded too tightly to the cardstock such that fragments of the paper substrate were torn off and retained on the tape surface (Figure 1B). These paper remnants would interfere with downstream purification steps and result in lower extraction yields.

Each of these conditions was difficult to control and resulted in variable or low sample yields. To circumvent these issues, skin tape discs were collected directly into microcentrifuge tubes with the adhesive side facing inward, thereby avoiding storage cards entirely (Figure 1C). This approach offers several benefits including (i) maintaining sterility and positive identification of each tape sample; (ii) retaining sample integrity, which enhances reproducibility for downstream proteomics and transcriptomics; and (iii) eliminating a downstream labor-intensive step, since transfer to microcentrifuge tubes is typically the first step before processing.

Another consideration at the site or collection stage is the total number of tapes to be collected. In this respect, we considered the patient experience for what was easily tolerated by the donor, as well as the required number or depth of collection for the downstream analysis of interest. Collection of biomarker samples with skin tapes is intended to be minimally invasive and appropriate for sensitive areas, longitudinal collections, and/or pediatric populations. Therefore, the primary collection should not result in pain, scarring, or any other disagreeable outcome. To determine the optimal number of tapes per collection time point, we tested ≤ 45 sequential tapes for redness and discomfort. Taking into account skin site, tape type, and individual variability, > 40 sequential tapes are often used to completely remove the stratum corneum for transepidermal penetration studies (29). However, this results in significant discomfort and “weeping” at the evaluation site due to the loss of the skin barrier. Our studies show that ≤ 20 tapes are well tolerated, are sufficient for most biomarker applications, and leave enough barrier intact to permit repeat collections with days or weeks (25, 30).

### 3.2 Optimizing extraction methods for protein and nucleic acids

**FIGURE 2:**
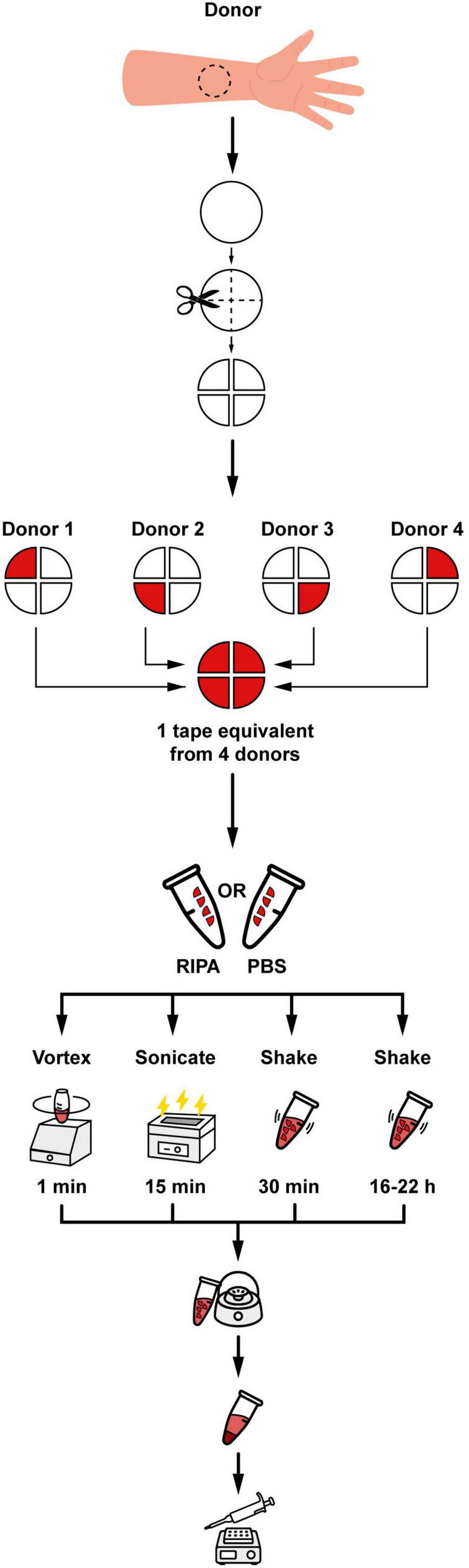
Ten skin tape disc samples collected from four healthy donors were used to evaluate different extraction methods. Five serial tapes were collected from two locations on the volar forearm. Each tape was quartered, and corresponding quarters were pooled across donors to minimize interindividual variability. For each condition, either PBS or RIPA buffer supplemented with 1 × protease/phosphatase inhibitor was added to a sample tube containing a pooled set of quarters. Samples were subjected to one of the following four lysis conditions (left to right): (1) vortexing for 1 min, (2) sonication for 15 min in an ultrasonic bath, (3) vigorous shaking at 4°C for 30 min, or (4) vigorous shaking at 4°C for 16–22 h. Samples were incubated on ice for 30 min prior to vortexing and sonication. After processing, samples were centrifuged, and the supernatant was collected to determine protein yield. PBS, phosphate-buffered saline; RIPA, radioimmunoprecipitation lysis and extraction buffer.

Following comprehensive review of the literature for previously published protein extraction methods, four lysis techniques and two buffer systems were selected for systematic comparative analysis. To minimize donor-specific variability, two sets of five sequential TS were collected from the volar forearm of healthy donors for a total of 10 tapes per donor. Each tape was then quartered and corresponding quarters were pooled across donors. Thus, the biologic sample compared across each lysis technique was nearly identical. The protein extraction methods (four lysis techniques and two buffer systems) were then systemically evaluated for total protein yield. Lysis methods included incubation in ice followed by vortexing for 1 min, incubation on ice followed by sonication for 15 min, vigorous shaking for 30 min, and shaking overnight (Figure 2). For each condition, samples were suspended in either PBS or RIPA buffer as potential dissociation buffer mediums, both supplemented with protease and phosphatase inhibitors.

**FIGURE 3:**
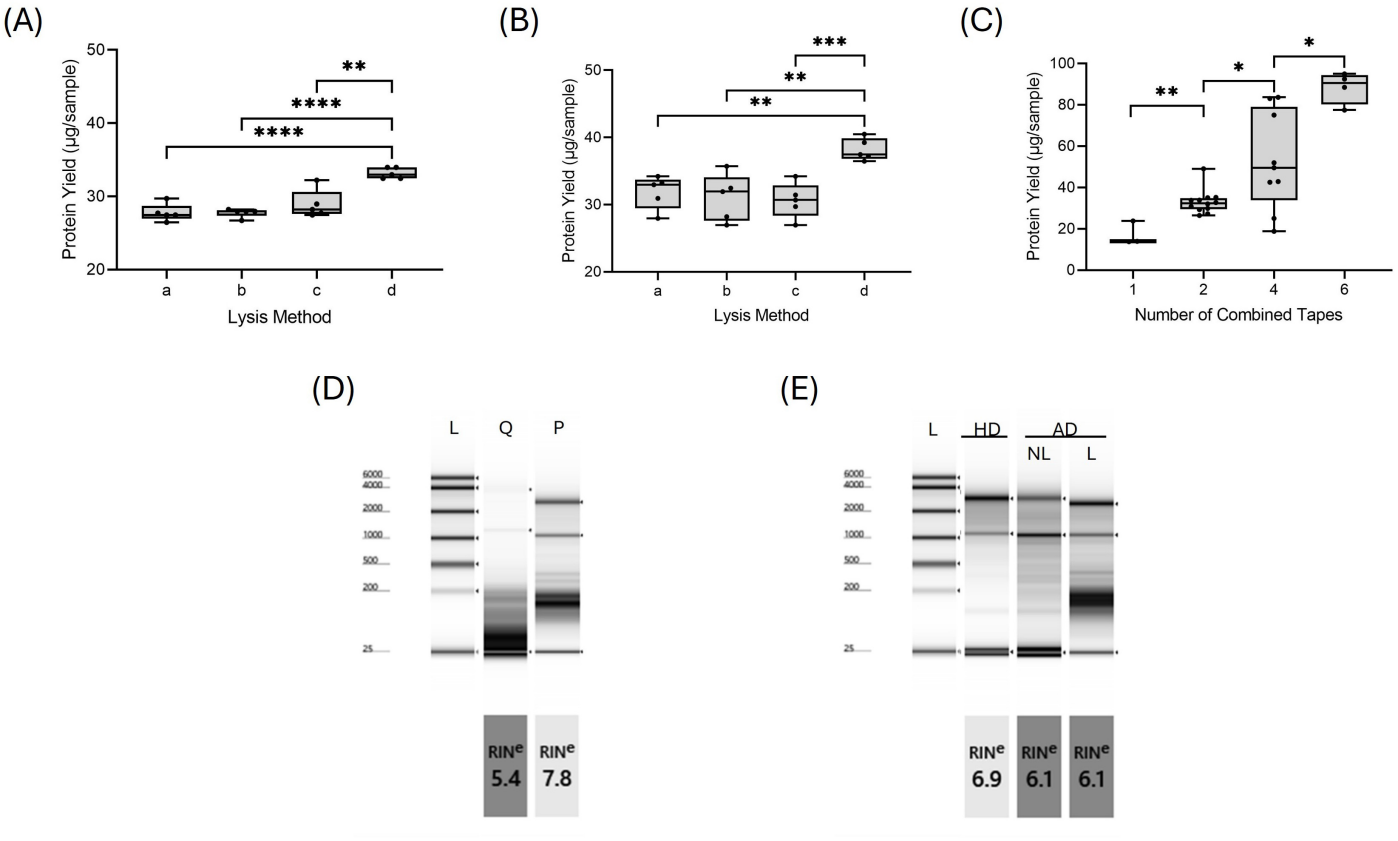
Comparison of protein yields using different lysis method parameters and RNA yields and quality metrics for RNA isolated from skin tapes. Samples were lysed in either PBS **(A)** or RIPA buffer **(B)** using one of the following mechanical dissociation methods: (a) brief vortexing, (b) 15-min sonication in an iced ultrasonic bath, (c) 30-min shaking at 4°C, and (d) overnight shaking at 4°C. Data points represent a single sample consisting of a pooled set of quarters. **(C)** Protein yield when harvesting multiple tapes together. **(D)** Microfluidic gel analysis using Agilent TapeStation depicting quality of RNA extracted by either a phenol-based method (Q) or the Promega method (P). **(E)** Representative gel images of RNA extracted using the Promega method from HD, AD NL, and AD L donors. Samples show distinct 18S and 28S ribosomal bands, consistent with the high RNA integrity scores. *P < 0.05; **P < 0.01; ***P < 0.001; ****P < 0.0001. AD, atopic dermatitis; HD, healthy donor; L, lesional; NL, nonlesional; PBS, phosphate-buffered saline; RIN, RNA integrity number; RIPA, radioimmunoprecipitation lysis and extraction buffer.

Prolonged shaking at 4°C (16–22 h) resulted in roughly 5 μg/tape greater overall protein yield than any other condition tested regardless of the lysis buffer: 33.2 ± 0.76 μg/tape in PBS and 38.2 ± 1.65 μg/tape in RIPA buffer (Figures 3A,B). No differences were observed among the short-duration methods within each buffer group. Protein extraction in RIPA buffer consistently outperformed extraction in PBS alone, although this difference was minor and suggests that PBS remains a viable option should downstream analyses require a detergent-free option. For all subsequent protein extraction methods reported herein, prolonged shaking at 4°C in a detergent-containing lysis buffer was adopted as the standard process.

Following this, scraping of sample tape discs with a rubber scraper (CELLTREAT Scientific Products) was added to the procedure to allow for pooling of multiple tape discs in minimal buffer volumes. The efficient removal of biological material from the tapes’ surface using a rubber scraper was confirmed by measuring the optical absorption (31). There was a ≥ 70% reduction in the observed amount of adhered biologic material after using the rubber scraper, indicating substantial recovery with this method. Pooling of 2–4 tapes yielded sufficient protein for most downstream workflows, including MSD, Olink, and MS-based approaches (Figure 3C). For other approaches, such as Western blot analyses, it may be necessary to pool ≥ 6–8 tapes to achieve higher protein concentrations.

Analogous to our approach for protein extraction from TS sampling discs, several RNA extraction methods were tested in house to determine which method would allow for highest yields without compromising sample quality. To circumvent variability due to biological sampling, multiple TS from a single donor were used to compare the following RNA extraction methods: (a) phenol-based extraction using QIAzol followed by column purification (Qiagen, RNeasy Mini Kit, Hilden, Germany), (b) automated bead-based extraction and purification using the Maxwell RSC Instrument (Promega, Madison, WI, United States), or (c) lysis in Buffer RLT, containing guanidine thiocyanate, followed by column-based purification (Qiagen, RNeasy Mini Kit, Hilden, Germany). Regardless of the extraction method used, batching multiple tapes into lots of ≥ 5 tapes was required to obtain sufficient RNA for downstream applications (i.e., < 5 tape sampling discs often resulted in RNA yields below the detection limit of the Qubit HS RNA Assay and produced no detectable bands in the Agilent TapeStation HS RNA ScreenTape Assay). The use of a rubber scraper for RNA extraction has previously been reported to enhance recovery of nucleic acid from skin tape samples (6). We employed a combination of techniques: tapes were first vortexed briefly in extraction buffer to dislodge the majority of biologic material, followed by a “clean-up” step using a rubber scraper only when visible residue remained. This sequential approach reduced adhesive carryover compared with scraping alone and ensured efficient transfer of biologic material. Up to 10 tapes per extraction were harvested in this manner to concentrate the sample into a single tube prior to initiating the extraction protocol. Combining 10 tapes per sample yielded sufficient RNA when extraction was performed with either traditional phenol-based or Promega kit lysis buffer. However, early pilot studies showed no appreciable yield using guanidine thiocyanate followed by column-based purification (not shown). In addition, automating the nucleic acid extraction process with the Promega Maxwell instrument allowed for greater numbers of samples to be processed per day. We opted to utilize this approach, prioritizing Promega’s published extraction methods. Optimal reproducibility was achieved using a modified version of Promega’s protocol utilizing the Maxwell RSC Blood DNA Kit for nucleic acid isolation from skin tape samples (Figures 3D,E). This method improved reproducibility and decreased hands-on time and therefore was adopted as the standard process for all subsequent RNA extraction discussed herein.

### 3.3 Protein and RNA recovery by disease state and sampling depth

**FIGURE 4:**
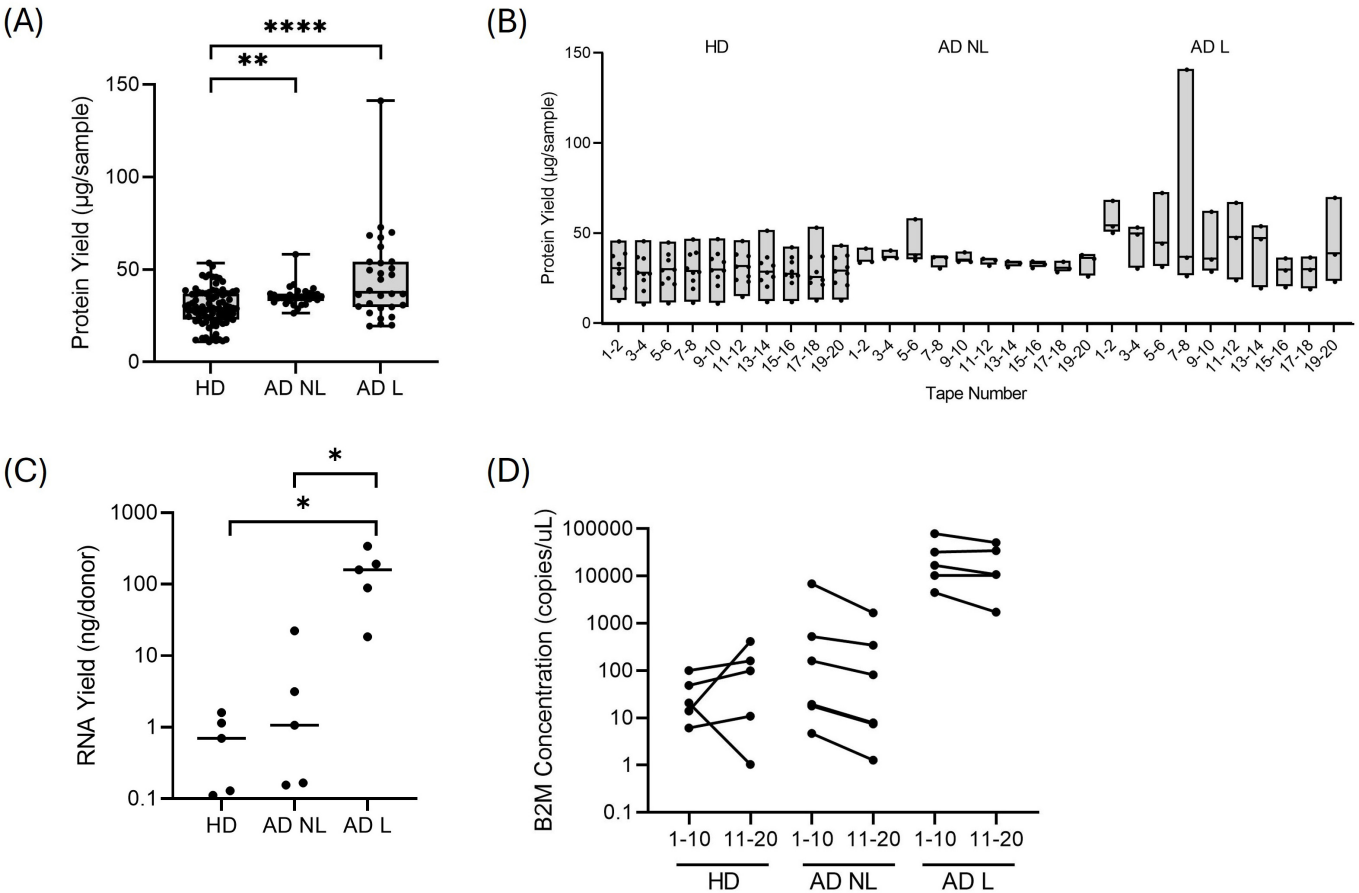
Comparison in protein yields from HD and AD tape strip samples. **(A)** Protein yield from skin tape samples collected from HD and AD donors. **(B)** Protein yield across the depth of the stratum corneum in HD and AD. Box plots show minimum and maximum and line depicts median. **(C)** Total RNA yield determined by ddPCR from pooled tape strips 1–20 for healthy and AD donors. **(D)** Levels of housekeeping gene B2M measured by droplet digital polymerase chain reaction. No significant difference in the expression levels of B2M was observed between tapes 1 to 10 and 11 to 20 within each group. *P < 0.05, **P < 0.01; ****P < 0.0001; AD, atopic dermatitis; B2M, β2-microglobulin; HD, healthy donor; L, lesional; NL, nonlesional.

We next sought to understand whether tape number (or sample depth) impacted the recoverable protein yield. TS samples were collected from 9 healthy donors and 3 AD donors (lesional and nonlesional skin). Samples were processed in successive batches of 2 tape discs each (e.g., 1–2, 3–4…), using the above protocol. Protein recovery was largely consistent across the sample tape numbers, regardless of skin source (healthy donors, AD lesional or nonlesional) (Figure 4A). AD lesional skin exhibited a pronounced degree of variability across the sampling depth; however, total protein yield was generally increased in AD lesional samples compared with healthy donors (Figure 4B). Next, the impact of tape number (or sample depth) on RNA yield was evaluated. For nucleic acid, a greater number of tape discs were required to obtain detectable RNA, thus tapes were separated into only 2 successive batches (i.e., 1–10 or 11–20). A large degree of donor-to-donor variability was observed; nonetheless, we found that RNA yield was generally greatest in AD lesional samples compared with that from healthy donors or nonlesional AD skin (Figures 4C,D). Further, the sample depth or tape number did not unambiguously lead to greater RNA yields, although a trend for greater yields in the more superficial tape batch (those containing tapes 1–10) was observed.

### 3.4 Superficial stratum corneum layers capture key inflammatory signals in AD

**FIGURE 5:**
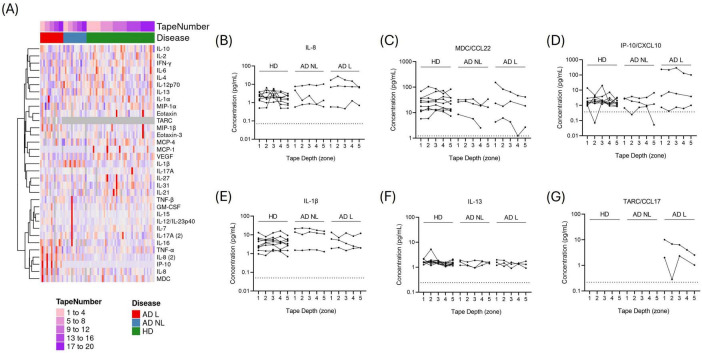
Protein isolated from 5 sets of 4 tapes representing various depths of collection from healthy (*n* = 9) and atopic dermatitis (*n* = 3) donors were tested in 4 MSD panels to determine the levels of cytokines across the depth of the stratum corneum. **(A)** Heat map showing the relative expression of cytokines measured by MSD. Expression values were log-transformed and normalized by Z-score across rows. **(B–G)** Line graphs show the concentrations measured in pg/mL for each patient across the depth of sampling. Tapes are broken into the following groups with zones 1–5 representing tapes 1–4, 5–8, 9–12, 13–16, and 17–20. AD, atopic dermatitis; CCL, C-C motif chemokine ligand; CXCL, C-X-C motif chemokine ligand; HD, healthy donor; IP-10, interferon gamma–inducible protein 10; IL interleukin; L, lesional; MDC, macrophage-derived chemokine; MSD, Meso Scale Discovery; NL, nonlesional; TARC, thymus and activation-regulated chemokine.

Given that total yield did not differ according to tape number or sample depth, we next compared the expression of various protein biomarkers to determine if expression varied by sample depth. Protein extracts from healthy or AD donors were pooled according to tape number into groups of 4 tapes each (i.e., tapes 1–4, 5–8, 9–12, 13–16, and 17–20). Each set was normalized to the same protein concentration and assessed by MSD for specific proinflammatory biomarkers (Figure 5). Most analytes exhibited low and relatively similar levels of expression in all groups with measurable, sometimes optimal, expression in the most superficial tape samples (tapes 1–4 and 5–8). For instance, chemokines and innate mediators such as macrophage inflammatory protein 1α (MIP-1α), IL-8, and IL-1β, as well as Th1-associated cytokines interferon gamma–inducible protein 10 (IP-10), and tumor necrosis factor α (TNF-α), were easily detected in samples composed of tape 1–4 and 5–8 (Figure 5A). These analytes are known to promote immune cell recruitment and increase inflammatory responses in AD (32–34) This is evidence that key inflammatory mediators in AD are quantifiable in the uppermost stratum corneum layers. Equally important in this analysis, all analytes quantifiable in later tape sample groups (i.e., tapes 13–16 and 17–20) were also measurable in more superficial groups (tapes 1–4 and 5–8), often at similar or higher levels.

MSD is a valuable technique for multianalyte panels. Nonetheless, we wished to confirm the validity of our extraction method for use with large-scale proteomic approaches, which are becoming more common for biomarker discovery since technologic improvements have enabled use with small input sample volumes. Two complementary technologies were explored for analysis following protein extraction from skin TS samples, both of which offer high sensitivity for low-abundance analytes: Olink Explore HT and LC-MS/MS. Previous reports have identified key mediators involved in the pathogenesis of AD using the Olink platform with skin TS samples (27). To our knowledge, LC-MS/MS-based technologies have had a more limited use with TS samples compared with immunoassays. However, LC-MS/MS–based technologies are able to identify structural proteins [e.g., FLG, keratins, involucrin (IVL)], enzymes (e.g., proteases), and insoluble barrier components (e.g., corneodesmosin, loricrin) that can reflect barrier integrity, desquamation, pH homeostasis, and inflammation (14, 35, 36). Thus, LC-MS/MS may be a particularly valuable technology for protein analysis in the context of skin disease and barrier-related diseases such as AD.

**FIGURE 6:**
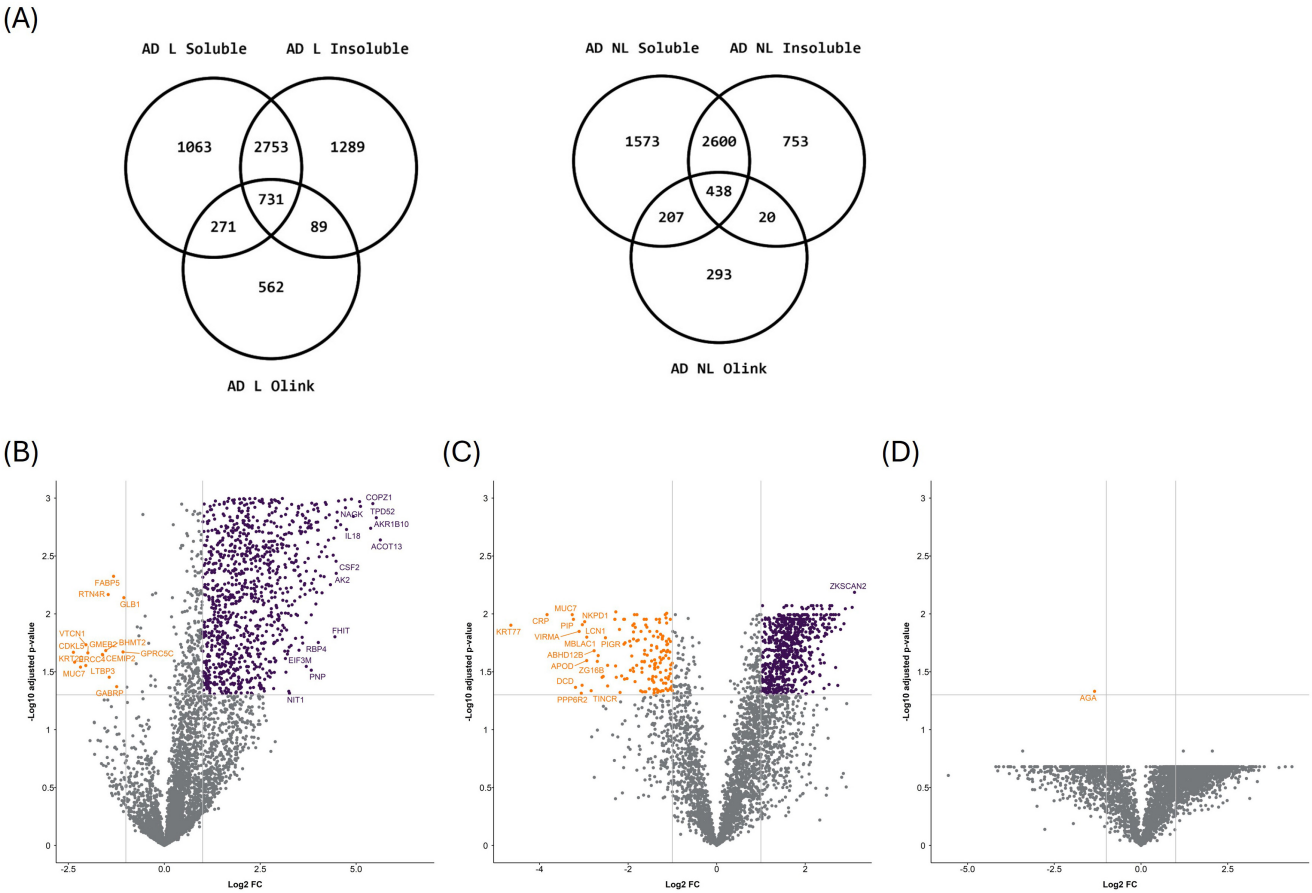
Proteomics analysis of skin tapes from a single AD donor in both L and NL samples. **(A)** Overlap of identified protein groups by the 3 discovery-based approaches. AD L and NL soluble represents LC-MS/MS data of peptides prepared using iST kits, AD L and NL insoluble represents LC-MS/MS data of peptides prepared using STraps, and AD L and NL Olink represents Explore HT data above the limit of detection. Volcano plots depict the proteins found to be differentially expressed between L and NL samples from a single AD donor from the **(B)** Olink Explore HT assay, **(C)** LC-MS/MS of insoluble proteins, and **(D)** LC-MS/MS of soluble proteins. AD, atopic dermatitis; L, lesional; LC-MS/MS, liquid chromatography tandem mass spectrometry; NL, nonlesional.

For discovery analyses, a single patient was tested using both lesional and nonlesional samples. The total measured proteins ranged from 5,884 protein groups in nonlesional to 6,758 protein groups in the lesional samples. Figure 6A shows the overlap of identified protein groups by the 3 discovery-based approaches on a lesional and nonlesional AD sample (Olink Explore HT and LC-MS/MS on both soluble and insoluble portions of the skin tape extracts). These data present very little overlap from the three approaches, with only approximately 10% of proteins measured in all three approaches, supporting the value of multiple approaches for discovery-based proteomics. Figures 6B–D present a single patient with nonlesional and lesional sample sites compared using a paired *t-*test and visualized as volcano plots with panel B representing Olink Explore HT, panel C representing LC-MS/MS of the insoluble pellet, and panel D representing LC-MS/MS of the soluble proteins. There were a total of 2,211 protein groups that were significantly (adjusted *P* < 0.05) greater in the lesional sample. Of these, only 164 were detected by both Olink Explore HT and LC-MS/MS, highlighting the complementary nature of these two approaches for biomarker discovery.

Many of the proteins found to be differentially expressed in our TS dataset have been previously detected in full-thickness skin biopsies and have known involvement in the pathogenesis of AD. LC-MS/MS revealed alterations in proteins related to barrier integrity (FLG, IVL, LOR), inflammatory keratins [keratin 16 (KRT16), keratin 6A (KRT6A)], innate immune activation (S100A8, S100A9), and oxidative stress [aldehyde dehydrogenase 3 family member A1 (ALDH3A1)], consistent with the chronic epithelial stress and impaired barrier function characteristic of AD (37–39). In parallel, Olink Explore HT detected increased levels of key type 2 immune mediators (CCL13, CCL17, CCL18, CCL22, IL-22, IL-18), neutrophil-attracting chemokines [CXCL (C-X-C motif chemokine ligand) 1, CXCL8], epithelial stress marker [amphiregulin (AREG)], and the tissue-remodeling enzyme [matrix metalloproteinase-9 (MMP9)] (37, 39–41). This further reinforces the value of integrating TS for the detection of key molecular mediators, while also demonstrating the utility of combining targeted and untargeted proteomic platforms for biomarker discovery.

### 3.5 RNA-seq analysis for AD gene signature

**FIGURE 7:**
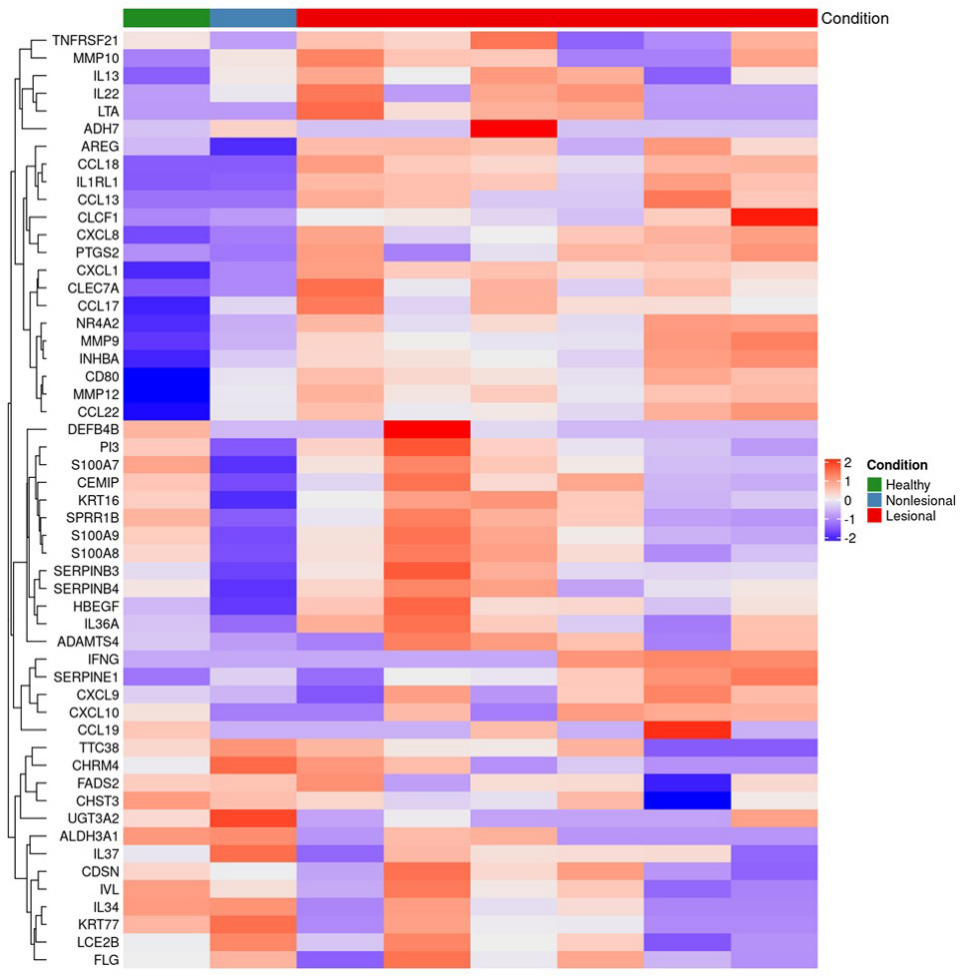
Heatmap showing relative gene expression across healthy, nonlesional, and lesional donors. RNA-seq was performed on pooled healthy and nonlesional samples and individual lesional samples. Expression values are row-wise Z-scores for selected immune, barrier, and inflammatory genes associated with atopic dermatitis.

To enable comparative transcriptomic profiling, RNA extracted from multiple donors was grouped by condition. Due to limited RNA recovery from individual tapes, samples from healthy donors and nonlesional AD sites were pooled to generate representative profiles for each group. In contrast, RNA yields from lesional AD samples were sufficient for individual library preparation. RNA-seq libraries were constructed and sequenced, and gene expression profiles were normalized and visualized as a heatmap (Figure 7), capturing the relative expression of key immune, barrier, and inflammatory genes across conditions.

The resulting expression patterns revealed a distinct AD-associated signature in lesional skin, characterized by increased expression of type 2 cytokines (e.g., IL-13, IL-22), chemokines (CCL17, CCL22, CXCL8), and tissue-remodeling enzyme (MMP9) (42–44). Upregulation of epithelial stress markers [AREG, heparin-binding epidermal growth factor–like growth factor (HBEGF), serpin family B member 3/4 (SERPINB3/B4)] and proinflammatory alarmins (S100A8, S100A9) further reflected ongoing inflammation in lesional skin (45–48). In contrast, structural proteins crucial to skin barrier integrity, such as FLG, IVL, and late cornified envelope 2B (LCE2B), were markedly downregulated in lesional skin of several donors compared with healthy or nonlesional samples (49). Together, these findings highlight a consistent lesional transcriptomic profile in AD, defined by barrier dysfunction, heightened type 2 inflammation, and immune activation.

## 4 Discussion

The integrity and utility of skin TS samples for downstream biomarker analyses are critically dependent on both the collection and handling protocols used. Our findings underscore the substantial variability introduced by traditional use of adhesive storage cards, which compromises both the retention of biological material and the reproducibility of analytical outcomes. Placing individual tape discs directly into microcentrifuge tubes with the adhesive surface oriented inward preserves sample identification, maintains sterility, and circumvents the need for labor-intensive sample transfers, offering a scalable, reproducible solution suited for multisite clinical trials.

Importantly, the donor experience was also a key consideration. Although 30–40 sequential tapes may be necessary for applications requiring the complete removal of the stratum corneum, our tolerance studies show that ≤ 20 tapes are sufficient for most biomarker analyses without inducing significant discomfort or barrier disruption. This makes the approach especially viable for pediatric populations and longitudinal studies.

Systematic evaluation of lysis conditions revealed that prolonged shaking at 4°C for 16–22 h yields the highest total protein recovery, with RIPA buffer slightly outperforming PBS. The addition of a rubber scraping step enhanced recovery and reproducibility, with higher overall protein concentrations especially in pooled samples. This strategy was thus incorporated as a standard step in all downstream workflows. Using this method, protein yields scaled predictably with the number of tapes pooled, supporting flexible adaptation of the protocol for high- and low-input applications. For example, 2–4 pooled tapes were sufficient for multiplex immunoassays and MS, while Western blotting may require larger pools.

**FIGURE 8:**
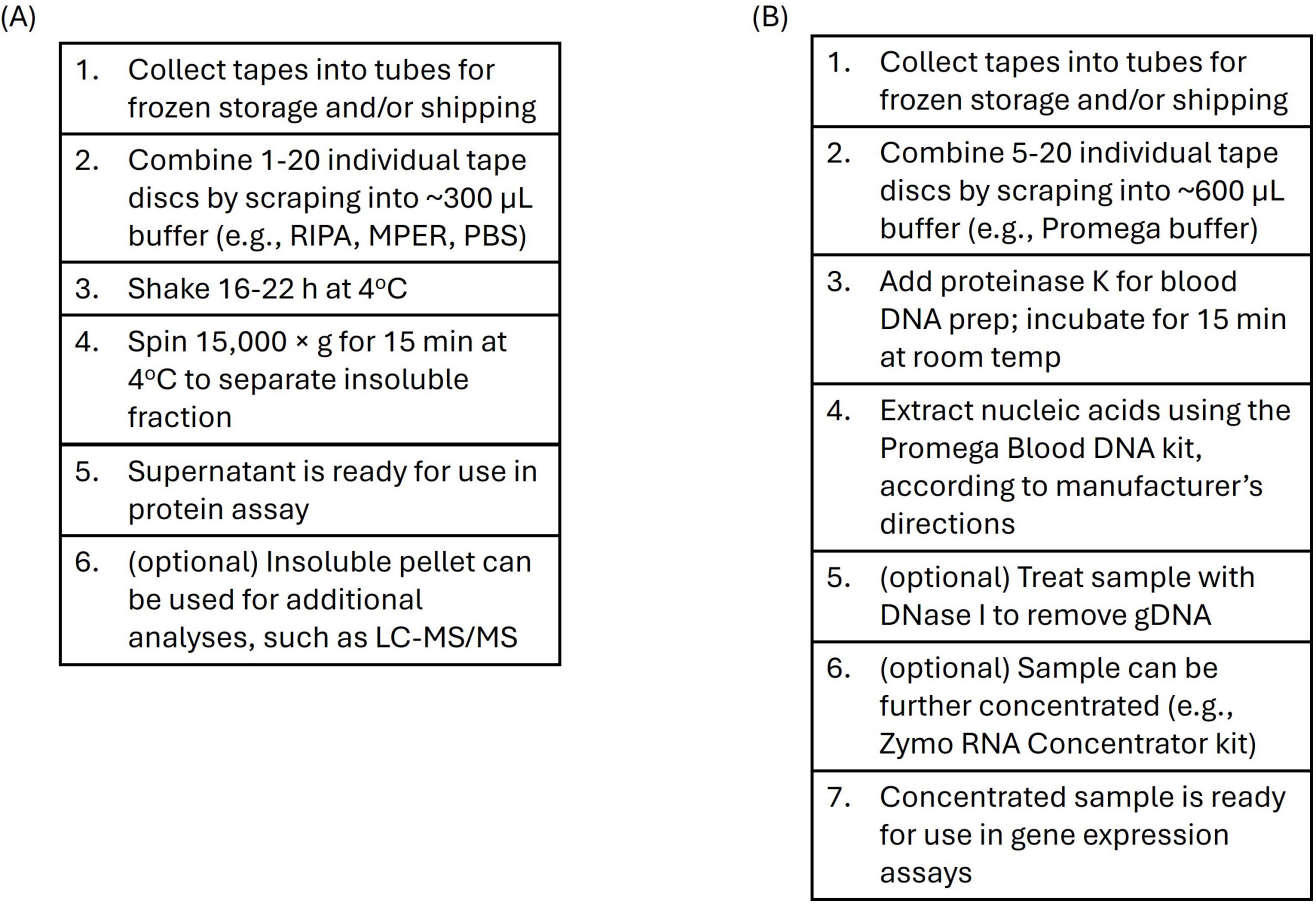
Protocol summary flow chart for isolation of both **(A)** protein and **(B)** nucleic acids from skin tape samples. DNase I, deoxyribonuclease I; LC-MS/MS, liquid chromatography tandem mass spectrometry; MPER, M-PER Mammalian Protein Extraction Reagent.

In parallel, RNA extraction protocols were evaluated using a similar pooled-tape, cross-donor normalization strategy to mitigate biological variability. The Promega Maxwell platform, with minor protocol optimizations, demonstrated superior reproducibility, reduced hands-on time, and higher RNA integrity (RIN values) compared with traditional phenol-based methods. Notably, similar to phenol-based methods, the Maxwell RSC Blood DNA kit allows for co-elution of both RNA and DNA from a single extraction protocol, offering flexibility for genomic and transcriptomic downstream applications. While the Maxwell RSC Blood DNA protocol was adopted for all RNA extractions in this study, a head-to-head comparison with the Maxwell simplyRNA Cells Kit showed comparable RNA yields and RIN values, with the added benefit of on-board DNase treatment, indicating its potential for further optimization and future use in TS-based RNA workflows. The outcome of these efforts is a robust protein and nucleic acid extraction protocol for skin tape strip sampling that can be broadly adapted to a wide range of downstream applications and is appropriate for use in large, multisite clinical trials (Figure 8).

Protein extractions revealed higher total yields from AD lesional samples compared with healthy or nonlesional skin, suggesting increased soluble protein content in inflamed AD skin. Likewise, RNA extraction followed a similar trend, with AD lesional samples yielding up to 340 ng of RNA, compared with 0.16–22 ng in nonlesional and < 1.61 ng in healthy skin. Whether these observations are broadly applicable to other skin conditions is not fully understood. However, it seems reasonable that rough, dry, or flaky skin has more surface area to adhere to tapes and therefore may supply greater biological material per tape disc compared with smooth, undamaged skin. For both RNA and protein extractions, the total yield was consistent across sampling depths or tape number, although a modest trend was observed for greater recovery from more superficial tapes. Further analyses focused on the spatial distribution of inflammatory biomarkers. Stratified grouping of TS revealed that key cytokines and chemokines, including IL-1β, IL-8, MIP-1α, IP-10, and TNF-α, were most abundant in the first few tapes collected (tapes 1–4 and 5–8). These findings reinforce previous reports that inflammatory signals are detectable in the superficial skin layers and support the use of minimally invasive TS as a viable method for capturing relevant immunologic activity in AD. Notably, analyte levels in deeper tape groups were never higher than those in superficial groups, suggesting no added biomarker benefit with deeper sampling and thus support methodology that requires < 20 consecutive tapes per sample for optimal balance of biomarker utility and donor tolerance.

The presented protein extraction method was also used to prepare samples for discovery-based proteomics analyses, which are commonly used for biomarker discovery. The soluble extracted proteins from 4 combined TS were analyzed using Olink Explore HT and both the soluble extracted proteins and the insoluble pellet were analyzed using LC-MS/MS. A single AD patient with both lesional and nonlesional samples was used for analysis and the multiproteomics approach measured 5,884 protein groups from nonlesional and 6,758 protein groups from lesional samples. These data support that skin tape samples are compatible with discovery proteomics approaches and that they can result in deep proteomic data for biomarker discovery. Such analyses present the ability to study a single patient in both lesional and adjacent nonlesional skin, presenting an opportunity to broaden understanding of diseases of the skin.

Consistent with the MSD findings, proteomic analyses using both LC-MS/MS and Olink revealed a robust AD-associated signature characterized by dysregulation of barrier-related proteins (e.g., FLG, KRT16), inflammatory mediators (e.g., S100A8, S100A9, IL-18), and type 2-associated cytokines and chemokines (e.g., CCL17, CCL22, IL-22). Transcriptomic profiling of lesional versus nonlesional and healthy samples further reinforced this signature, with increased expression of IL-13, IL-22, CCL17, and AREG and reduced expression of key structural genes such as FLG, IVL, and LCE2B. These data demonstrate that skin tape-derived samples not only yield sufficient material for high-throughput proteomics and RNA sequencing but also capture biologically meaningful, multi-omic signatures reflective of AD pathogenesis. Due to the technical nature of this body of work, AD samples were characterized simply as lesional or nonlesional and were not further stratified according to lesion severity or other clinical features [e.g., Eczema Area and Severity Index (EASI), itch numerical rating scale, TEWL]. As such, the relationship of TS-derived biomarkers to clinical features was not evaluated in this report. Nonetheless, such correlations have previously been shown with greater patient numbers (18, 50, 51). It follows that improved standardization of TS samples may serve to strengthen these correlations. Future studies will ultimately be needed to confirm the strength of clinical correlations with TS-derived biomarkers.

Collectively, these results inform critical operational decisions for implementing TS sampling in large-scale clinical studies. Optimized collection, storage, and extraction protocols enhance sample integrity and molecular yield and reduce variability introduced by donor discomfort and processing inconsistencies. Our data also inform areas to modify this procedure or extraction methods for alternate downstream approaches. For instance, we show that sufficient protein yield is possible with this method even if using a detergent-free extraction buffer. Taken together, the insights gained here—particularly around disease-state stratification and sampling depth—highlight the potential for skin TS sampling to capture biologically relevant signals in dermatologic diseases like AD. Future studies may build on these findings by integrating longitudinal sampling and expanding analyses to include other analyte types (e.g., lipids, microbiome, etc.) or other skin conditions. Notably, non- and minimally invasive technologies for skin biosample collection continue to advance within the field. Future clinical dermatology studies would benefit from direct comparisons of biomarker signatures collected via skin scraping, microneedle technologies and/or noninvasive imaging approaches; direct head-to-head comparisons have not yet been performed. Of these, TS currently has the broadest uptake for clinical trial and biomarker sampling usage based on the substantial number of times they are discussed in the literature. In contrast, although scraping is commonly used for diagnostic microscopy in dermatology practice, it is rarely described as a standard method for molecular biomarker analyses. Likewise, imaging and microneedle approaches remain in their infancy for widespread use as a clinical biomarker solution.

Employing the optimized TS sample workflow (Figure 8) will alleviate concerns about TS sample standardization or inconsistent yields. The methodology described is largely standard practice for a wide range of testing facilities or academic sites with regard to materials, buffers, and methods. We anticipate that greater standardization of implementation and processing procedures will lead to higher-quality data collection and ultimately result in improved drug development pathways.

## Data Availability

Original datasets presented in this study are available in a publicly accessible repository: https://www.ncbi.nlm.nih.gov/bioproject/PRJNA1463695 [ncbi.nlm.nih.gov].
